# Targeting mTOR dependency in pancreatic cancer

**DOI:** 10.1136/gutjnl-2013-306202

**Published:** 2014-04-09

**Authors:** Douglas C Morran, Jianmin Wu, Nigel B Jamieson, Agata Mrowinska, Gabriela Kalna, Saadia A Karim, Amy Y M Au, Christopher J Scarlett, David K Chang, Malgorzata Z Pajak, Australian Pancreatic Cancer Genome Initiative (APGI), Karin A Oien, Colin J McKay, C Ross Carter, Gerry Gillen, Sue Champion, Sally L Pimlott, Kurt I Anderson, T R Jeffry Evans, Sean M Grimmond, Andrew V Biankin, Owen J Sansom, Jennifer P Morton

**Affiliations:** 1 CRUK Beatson Institute, Glasgow, UK; 2 The Kinghorn Cancer Centre and the Cancer Research Program, Garvan Institute of Medical Research, Darlinghurst, Sydney, New South Wales, Australia; 3 West of Scotland Pancreatic Unit, Glasgow Royal Infirmary, Glasgow, UK; 4 School of Environmental & Life Sciences, University of Newcastle, Ourimbah, New South Wales, Australia; 5 Department of Surgery, Bankstown Hospital, Bankstown, Sydney, New South Wales, Australia; 6 Faculty of Medicine, South Western Sydney Clinical School, University of NSW, Liverpool, New South Wales, Australia; 7 The Wolfson Wohl Cancer Research Centre, Institute of Cancer Sciences, University of Glasgow, Glasgow, UK; 8 Institute of Cancer Sciences, College of Medical, Veterinary and Life Sciences, University of Glasgow, Glasgow, UK; 9 West of Scotland PET Centre, Gartnavel General Hospital, Glasgow, UK; 10 West of Scotland Radionuclide Dispensary, NHS Greater Glasgow and Clyde, Glasgow, UK; 11 Queensland Centre for Medical Genomics, Institute for Molecular Bioscience, University of Queensland, St Lucia, Brisbane, Queensland, Australia

**Keywords:** Pancreatic Cancer, Pharmacogenomics, Cell Signalling, Genetics

## Abstract

**Objective:**

Pancreatic cancer is a leading cause of cancer-related death in the Western world. Current chemotherapy regimens have modest survival benefit. Thus, novel, effective therapies are required for treatment of this disease.

**Design:**

Activating *KRAS* mutation almost always drives pancreatic tumour initiation, however, deregulation of other potentially druggable pathways promotes tumour progression. PTEN loss leads to acceleration of *Kras^G12D^*-driven pancreatic ductal adenocarcinoma (PDAC) in mice and these tumours have high levels of mammalian target of rapamycin (mTOR) signalling. To test whether these KRAS PTEN pancreatic tumours show mTOR dependence, we compared response to mTOR inhibition in this model, to the response in another established model of pancreatic cancer, KRAS P53. We also assessed whether there was a subset of pancreatic cancer patients who may respond to mTOR inhibition.

**Results:**

We found that tumours in KRAS PTEN mice exhibit a remarkable dependence on mTOR signalling. In these tumours, mTOR inhibition leads to proliferative arrest and even tumour regression. Further, we could measure response using clinically applicable positron emission tomography imaging. Importantly, pancreatic tumours driven by activated KRAS and mutant p53 did not respond to treatment. In human tumours, approximately 20% of cases demonstrated low PTEN expression and a gene expression signature that overlaps with murine KRAS PTEN tumours.

**Conclusions:**

KRAS PTEN tumours are uniquely responsive to mTOR inhibition. Targeted anti-mTOR therapies may offer clinical benefit in subsets of human PDAC selected based on genotype, that are dependent on mTOR signalling. Thus, the genetic signatures of human tumours could be used to direct pancreatic cancer treatment in the future.

**Significance of this study**

**What is already known on this subject?**

- Pancreatic cancer is one of the leading causes of cancer death. Most therapies are largely ineffective and new therapies are required.
- Pancreatic cancer is nearly always driven by KRAS mutation, with progression driven by mutations in other genes, notably CDKN2A, TP53 and DPC4. The disease is very complex genetically, however, and many more genes are mutated at low frequencies.
- There may be pathways that, although deregulated relatively rarely, are key to driving specific tumours.

**What are the new findings?**

- Mammalian target of rapamycin (mTOR) inhibition can lead to proliferative arrest and even tumour regression in pancreatic tumours driven by activated KRAS and PTEN deficiency, but not in tumours driven by activated KRAS and mutant p53.
- Therapeutic response to mTOR inhibition can be assessed using clinically applicable positron emission tomography imaging.
- ∼20% human pancreatic tumours exhibit low PTEN expression, and a gene expression signature that overlaps with murine KRAS PTEN tumours.

**How might it impact on clinical practice in the foreseeable future?**

- This study is important as it is the first to show efficacy of a targeted therapy in a preclinical model of pancreatic cancer using the genotype-to-phenotype approach.
- Targeted anti-mTOR therapies may offer clinical benefit in subsets of human pancreatic ductal adenocarcinoma, selected based on genotype that are dependent on mTOR signalling.
- The genetic signatures of human tumours could be used to direct personalised pancreatic cancer treatment in the future.

## Introduction

Pancreatic ductal adenocarcinoma (PDAC) is the fourth commonest cause of cancer death in the UK and has an estimated global incidence of 279 000 per annum.^1^ There has been minimal improvement in survival for over 30 years, and 80–90% of cases present with either locally advanced or metastatic disease, which precludes curative surgery. The majority of patients who do undergo resection inevitably develop recurrent or metastatic disease. Additionally, most systemic therapies are largely ineffective. Gemcitabine monotherapy has modest clinical benefit and a marginal survival advantage in patients with advanced PDAC,^2^ however, the median survival of patients with metastatic PDAC remains poor, and is often less than 6 months.^2^ More recently, encouraging results have been observed in clinical trials with the FOLRIFINOX regimen,^3^ although many patients are unable to tolerate this regimen. Consequently, novel, effective therapies are required for advanced and early disease.

PDAC development follows a well-characterised progression model from benign precursor lesions known as pancreatic epithelial neoplasia (PanIN) to the highly aggressive resultant tumour. In almost all cases, mutation of *KRAS* is the likely initiating lesion. The subsequent acquisition of mutations in a number of tumour suppressor genes, notably *CDKN2A*, *TP53* and *DPC4*, and many more at lower frequencies,^4^ leads to tumour progression and metastasis, in a process now believed to occur over a period of 10–20 years.^5^ Recent sequencing studies of pancreatic cancer have reinforced the complexity and heterogeneity of this disease.^4^ Thus, although there may be pathways that are key to driving specific tumours, they may be deregulated relatively rarely. Targeting the consequent aberrant signalling pathways, however, represents an attractive novel therapeutic approach in patients selected on their molecular profile.

This approach leads to challenges in recruiting adequate numbers of such patients for clinical studies. Here, preclinical mouse models provide the opportunity to identify key actionable phenotypes and distinct sensitivities, and build confidence in observations in very low patient numbers. In fact, two reports of exceptional responders to mammalian target of rapamycin (mTOR) inhibition were recently published, first, in a patient with Peutz–Jeghers syndrome and advanced pancreatic cancer,^6^ and second, in a single patient in a trial of an AKT inhibitor who was subsequently shown to have activating *KRAS* mutation and loss of *PTEN.*^7^ These studies suggest that there may be a therapeutic opportunity for inhibition of mTOR in selected patients with pancreatic cancer.

Recent clinical interest in the inhibition of mTOR has been renewed with the demonstration of antitumour activity in patients with metastatic renal cell carcinoma.^8^ By contrast, studies have failed to show antitumour efficacy in patients with pancreatic cancer.^9^ ^10^ However, these phase II clinical trials were performed in patients with gemcitabine-refractory disease, and with no patient selection based on the molecular pathology of the tumour. Pancreatic cancers are often described as heterogeneous and, while in excess of 90% will have activation of *KRAS*,^11^ this is invariably accompanied by a wide variety of tumour suppressor losses,^12^ which may contribute to the variability of clinical response to inhibition of specific pathways. We recently found that approximately 15–20% of human PDAC exhibit very high levels of active phosphorylated mTOR^S2448^, and these patients have significantly reduced survival. Although not typically mutated in pancreatic cancer, *Pten* was highly mutated in two recent screens for gene mutations that accelerate Kras^G12D^-driven pancreatic tumorigenesis.^13^ ^14^ Additionally, we, and others, developed a mouse model in which pancreas specific deletion of one copy of PTEN, the negative regulator of mTOR, rapidly accelerated Kras^G12D^-driven PDAC.^15^ ^16^

In this study, we show that murine pancreatic tumours driven by activated *Kras* and *Pten* deficiency are highly sensitive to mTOR inhibition, by contrast with tumours driven by activated *Kras* and mutation of *Trp53*, demonstrating that the therapeutic ‘phenotype’ is dependent on the genotype of tumours. Further, we show that PTEN-deficient tumours regress upon treatment, and undergo a proliferative arrest that can be monitored using positron emission tomography (PET) CT imaging, thus providing a clinically relevant functional biomarker of therapeutic efficacy. Genetically engineered mouse models, such as these, are particularly useful to study PDAC and test novel therapies, given that they closely recapitulate the human disease. In the future, it is likely that they will become more widely used preclinically to better model genotype-to-phenotype approaches.

## Methods

### Genetically modified mice

The *Pdx1-Cre*, *LSL-Kras^G12D^*, *Pten^fl^*, and *LSL-Trp53^R172H^* mice have been described previously.^15^ ^17^ ^18^ Mice on a mixed strain background were kept in conventional animal facilities and experiments carried out in compliance with UK Home Office guidelines. Mice were genotyped by Transnetyx (Cordova, Tennessee, USA).

Mice were treated with 10 mg/kg rapamycin or vehicle daily by intraperitoneal injection, and/or 100 mg/kg gemcitabine twice weekly by intraperitoneal injection. Animals were sacrificed as per institutional guidelines, and tissues removed and fixed in 10% buffered formalin.

### Ultrasound imaging

High-resolution ultrasound imaging was performed using the Vevo770 System with a 35 MHz Real-Time Micro Visualisation (RMV) scanhead (VisualSonics) as described previously.^19^ Tumours were measured from two dimensional images at the maximal dimensions of the tumour. Anaesthesia was induced and maintained throughout the procedure with a mixture of isoflurane and medical air.

### ^18^F-3′-Fluoro-3′-deoxy-L-Thymidine PET-CT imaging

Pretreatment and post-treatment with rapamycin, mice were anesthetised and given an intravenous bolus of ^18^F-3′-Fluoro-3′-deoxy-L-Thymidine (^18^F-FLT, ∼6 MBq). After an uptake phase of 2 h, PET-CT images were acquired using an Albira scanner (Bruker, Billerica, Massachusetts, USA). Further details are provided in the online supplementary material.

### Immunohistochemistry

Immunohistochemical (IHC) analysis was performed on formalin-fixed paraffin-embedded sections according to standard protocols. Primary antibodies used were anti-Pten, 1:100, anti-pAkt^S473^ (1:50), anti-pmTOR^S2448^ (1:100), anti-pS6 (1:400), anti-4EBP1 (1:500) (all Cell Signalling Technology), anti-Ki67 (1:200), anti-p53 (1:200), anti-CD3 (1:75) (all Vector), anticleaved caspase 3 (1:800, R&D) and anti-CD31 (1:100, Abcam).

### Tumour cell lines

Isolation of mouse PDAC cell lines from KC PTEN and KPC has been previously described.^15^ ^17^ Cell lines were cultured in Dulbecco's modified Eagle's medium (Invitrogen) supplemented with 10% FBS, 2 mM L-glutamine (Invitrogen) and penicillin/streptomycin (50 units/mL) (Invitrogen), in a humidified incubator at 37°C.

### Immunoblotting

Western immunoblotting was performed according to standard protocols. Primary antibodies used were against S6, pS6^S235/236^, Akt, pAkt^S473^, mTOR, pmTOR^S2448^ (all 1:1000, Cell Signalling Technology), and β-actin (1:5000, Sigma–Aldrich).

### Tissue microarray analysis

The Glasgow human pancreatico-biliary tissue microarray has been described previously.^20^ PTEN expression levels were scored based on staining intensity and area of tumour using a weighted histoscore: Σ(1×%weak)+(2×%moderate)+(3×%strong). Kaplan–Meier survival analysis with Log-Rank statistical test was used to analyse overall survival from time of surgery. All statistical analyses were performed using SPSS V.19 (Chicago, Illinois, USA).

### Gene expression analysis and signature generation

RNA was isolated from mouse tumours using the RNeasy mini kit (Qiagen). At least three mice of each genotype were arrayed on Affymetrix microarrays (Paterson Institute Microarray Service). The affymetrix cell intensity (cel) files were normalised with Robust Multiarray Analysis in Partek Genomics Suite Software. Anova was used to identify significantly regulated genes and linear contrasts calculated between all pairs of experimental groups. Multiple test correction was performed for all calculated p values employing Benjamini and Hochberg's step-up method. Further details are provided in the online supplementary material.

## Results

### mTOR inhibition improves survival in a mouse model of PTEN-deficient PDAC

Genetically engineered mouse models (GEMMs) of PDAC recapitulate human pancreatic cancer in a number of ways, including in their resistance to standard therapies.^19^ Thus, we used GEMMs to assess whether tumours with activation of the mTOR pathway would be exquisitely sensitive to mTOR inhibition. The foundation for these models was the *Pdx1-Cre*; *Kras^G12D/+^* (KC) mouse model, in which expression of activated Kras is targeted to the mouse pancreas using a conditional *LSL-Kras^G12D^* allele activated by Cre-mediated recombination, with Cre under the control of the pancreatic and duodenal homeobox1 promoter (Pdx1). These KC mice develop PanINs throughout their pancreas, which appear largely senescent,^17^ but progress to develop invasive PDAC at low frequency and with prolonged latency.^21^ When KC mice are crossed with animals bearing a *Pten* allele flanked by *loxP* sites, to generate *Pdx1-Cre*; *Kras^G12D/+^*; *Pten*^flox/+^ (KC PTEN) mice, tumorigenesis is rapidly accelerated.^15^

As we wanted to determine whether mTOR inhibition with rapamycin could arrest tumour growth in mice with late-stage disease, we compared treatment of KC PTEN mice, with treatment in *Pdx1-Cre*; *Kras^G12D/+^*; *Trp53^R172H/+^* (KPC) mice,^18^ which are resistant to most therapies.^19^ Cohorts of KC PTEN and KPC mice were established, and animals monitored until they developed clinically detectable pancreatic tumours, at which point mice would normally be sacrificed within 1–3 days. Clinical features displayed by these mice include abdominal distension with a palpable mass, weight loss and reduced mobility. At this stage, mice were examined by ultrasound imaging to confirm the presence of pancreatic tumour, and to assess tumour size. Mice were treated with rapamycin, vehicle control, gemcitabine, or rapamycin in combination with gemcitabine, monitored daily for clinical signs, and euthanased when symptoms worsened.

**Figure 1:**
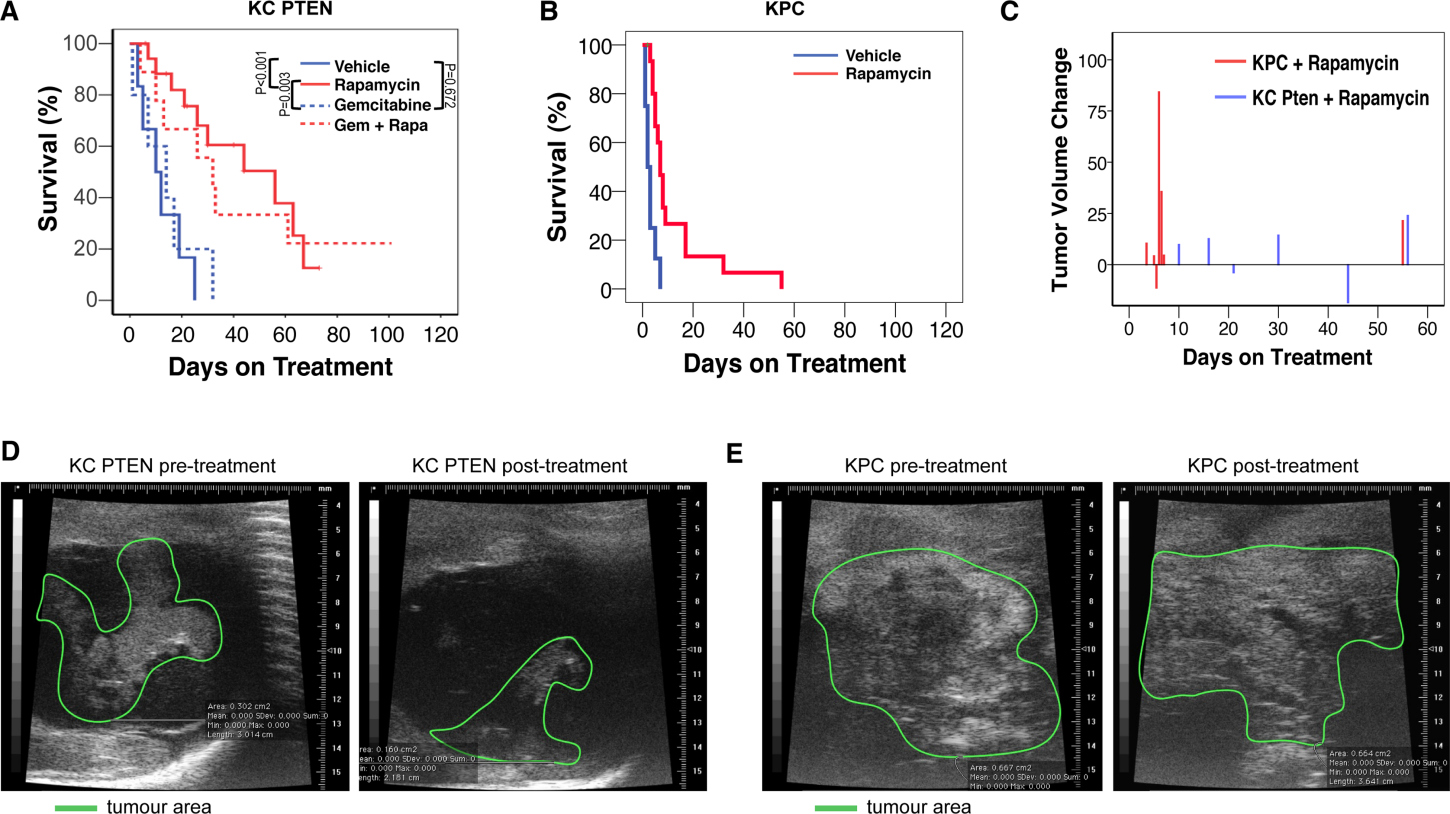
Inhibition of mammalian target of rapamycin (mTOR) can delay tumorigenesis and improve survival even in late-stage PTEN-deficient pancreatic ductal adenocarcinoma (PDAC). (A) Kaplan–Meier survival curve showing that the survival of KC PTEN mice with symptomatic PDAC treated daily with either 10 mg/kg intraperitoneal rapamycin as a single agent (n=18, red solid line), or in combination with twice weekly 100 mg/kg intraperitoneal gemcitabine (n=9, red dashed line), was significantly increased compared with either vehicle control treated mice (n=6, blue solid line), or with gemcitabine treated mice (n=5, blue dashed line). (B) Kaplan–Meier survival curve showing that the survival of KPC mice with symptomatic PDAC treated daily with 10 mg/kg intraperitoneal rapamycin (n=16, red line), was not significantly increased compared with vehicle control treated mice (n=8, blue solid line). (C) Chart showing the change in tumour volume between the start of rapamycin treatment and the time of sacrifice (days of treatment on x-axis) in KC PTEN mice (blue bars) compared with KPC mice (red bars). (D) Ultrasound images of a pancreatic tumour in a KC PTEN mouse prior to and post-treatment. (E) Ultrasound images of a pancreatic tumour in a KPC mouse prior to and post-treatment.

In KC PTEN mice, rapamycin treatment either alone, or in combination with gemcitabine (median survival, 56 days and 32 days, respectively) resulted in significant clinical improvement and a clear survival advantage compared with vehicle-treated controls or gemcitabine monotherapy (median survival, 10 days and 14 days, respectively) (figure 1A). Gemcitabine monotherapy had negligible benefit, in line with recent studies,^19^ ^22^ ^23^ and the increased survival in response to rapamycin therapy was not improved by combination with gemcitabine (figure 1A). Importantly, by contrast with the significant survival benefit observed in KC PTEN mice, rapamycin treatment offered little clinical benefit in KPC mice (figure 1B), and the improvement in survival was negligible compared with vehicle-treated controls (median survival, 7days vs 2 days). Thus, treatment with rapamycin is only effective in PTEN deficient tumours, suggesting that tumours with deregulated mTOR signalling may be uniquely sensitive to mTOR inhibition.

### mTOR inhibition can induce tumour shrinkage in Pten-deficient PDAC

In order to confirm presence of tumour in symptomatic animals prior to treatment, and to assess response, in terms of size, to rapamycin or vehicle, we used in vivo ultrasound imaging. Initial measurements of maximal tumour cross-sectional area were on the first day of treatment. When disease progressed, animals were imaged again and a further cross-sectional measurement taken of the tumour (figure 1C–E). We observed tumour shrinkage in several of the KC PTEN mice following rapamycin treatment (figure 1C,D), and even in those KC PTEN tumours that did not regress, little progression was observed even over several weeks of follow-up. By contrast, we failed to achieve significant responses in any KPC mice following rapamycin treatment, and all KPC tumours showed a steady increase in size, even over a brief time period, as symptoms quickly worsened (figure 1C,E).

**Figure 2:**
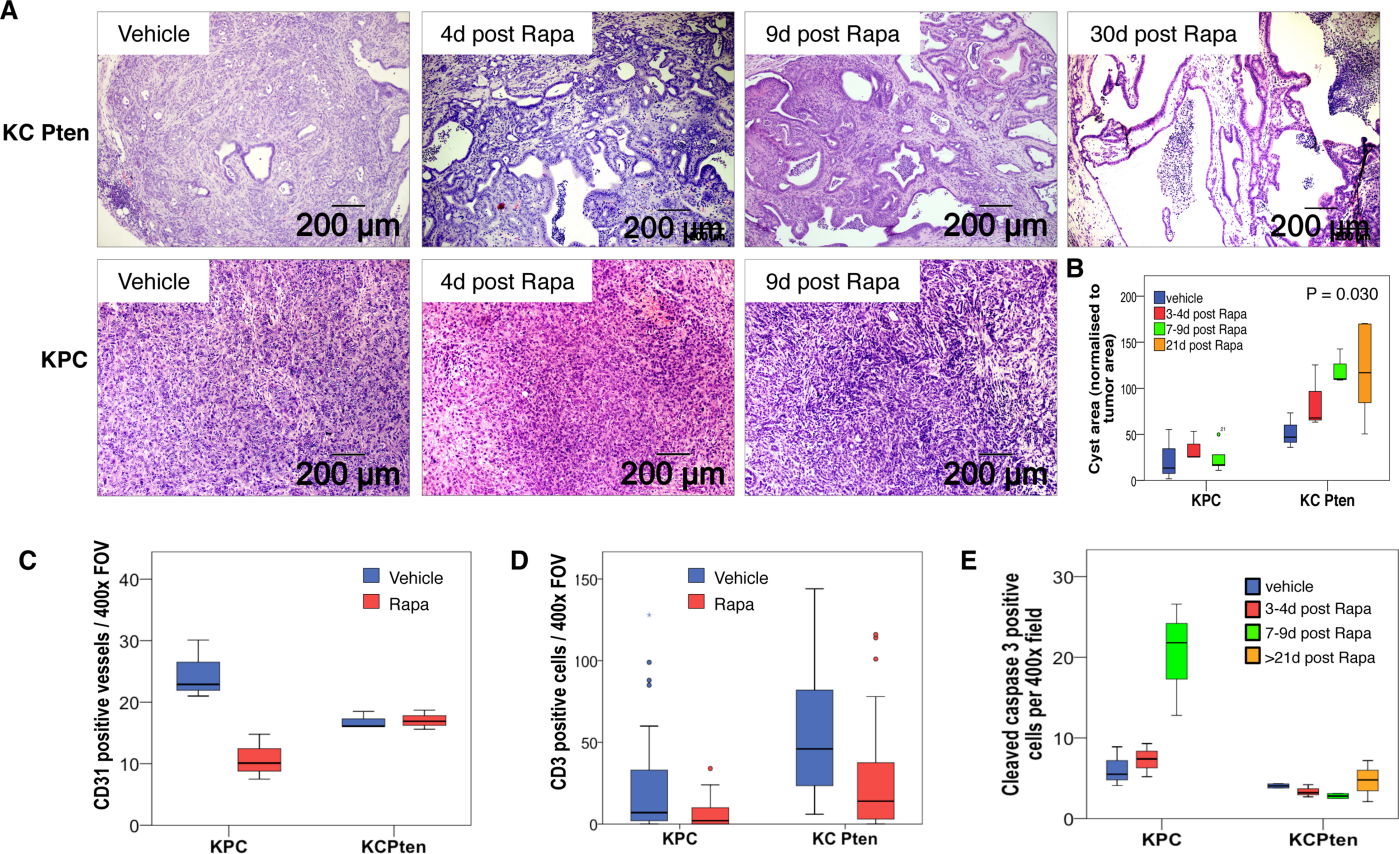
Mammalian target of rapamycin (mTOR) inhibition can induce tumour regression in Pten-deficient pancreatic ductal adenocarcinoma (PDAC). (A) H&E-stained sections of PDAC harvested from mice treated with vehicle or 10 mg/kg rapamycin for 4, 9 or 30 days, as indicated. Cyst formation is observed, and increases with time on treatment in KC PTEN mice (upper panels), but not in KPC mice (lower panels). (B) Boxplot showing quantification of cyst area as normalised to the total tumour area. (C) Boxplot showing quantification of the number of CD31-positive vessels per 400× field of view in sections from rapamycin treated (red bars) or vehicle treated (blue bars) KC PTEN or KPC mice, as indicated. (D) Boxplot showing quantification of the number of CD3 positive cells per 400× field of view in sections from rapamycin treated (red bars) or vehicle treated (blue bars) KC PTEN or KPC mice, as indicated. (E) Graph showing quantification of the number of cleaved caspase 3 positive cells per 400× field of view in sections from rapamycin, or vehicle treated KC PTEN or KPC mice, as indicated (blue=vehicle, red=3–4 days rapamycin, green=7–9 days rapamycin, orange=>21 days rapamycin). 10 fields were assessed per mouse and at least three mice for each treatment group.

When the pancreata of mice were examined histologically, we observed marked changes in rapamycin-treated KC PTEN mice that became more pronounced with time on treatment. Solid tumour tissue appeared to regress, causing the formation of cysts within the pancreas (figure 2A). Importantly, neither vehicle-treated tumours (figure 2A, left panels), nor rapamycin-treated KPC tumours (figure 2A, lower panels,) were affected histologically. The area of cystic morphology was significantly higher in rapamycin-treated KC PTEN tumours compared with KPC tumours (figure 2B, p=0.030), and correlated with duration of treatment (figure 2B, Spearman's r=0.596, p=0.019). Our data suggest that mTOR inhibition may be effective in a subset of human pancreatic tumours that are dependent on mTOR signalling, and importantly, could offer clinical benefit even in patients with late-stage disease.

Rapamycin has been reported to have antiangiogenic effects,^24^ so we performed IHC staining for the endothelial cell marker CD31, to allow us to score vasculature in treated mice. Importantly, we found that rapamycin treatment had no discernable effect on the vasculature in KC PTEN mice following treatment, although there was a significant reduction in vessel counts in the KPC mouse model (p=0.050, figure 2C). Thus, rapamycin does not exert its antitumoral effect through inhibiting angiogenesis. Rapamycin also has immunosuppressive effects, particularly targeting T cells,^25^ so we also examined the number of CD3-positive T cells in the pancreata of our mice following treatment. We were able to observe a marked reduction in the number of CD3-positive T-cells in response to rapamycin treatment, however, this was equivalent in the KC PTEN and KPC mice (figure 2D). These data suggest that rapamycin is not exerting its effect via suppression of T-cells, and our data thus far implies a direct targeting of tumour cells.

### mTOR inhibition abrogates proliferation in Pten-deficient PDAC

We were therefore interested in understanding how mTOR inhibition affected tumours in KC PTEN mice at cellular level. To test whether rapamycin could induce apoptosis of tumour cells, we performed IHC for cleaved caspase 3. Tumours harvested from mice 3–4, 7–9 and >21 days post-treatment were assessed, and we found that there was no significant induction of apoptosis in response to rapamycin treatment in KC PTEN mice (figure 2E). There was no significant induction of apoptosis in KPC mice 3–4 days after treatment either, but in those mice that survived 7–9 days post-treatment there was an increase (p=0.050) in apoptotic cells, potentially due to the size of tumours, and resulting hypoxia and necrosis by this time-point.

**Figure 3:**
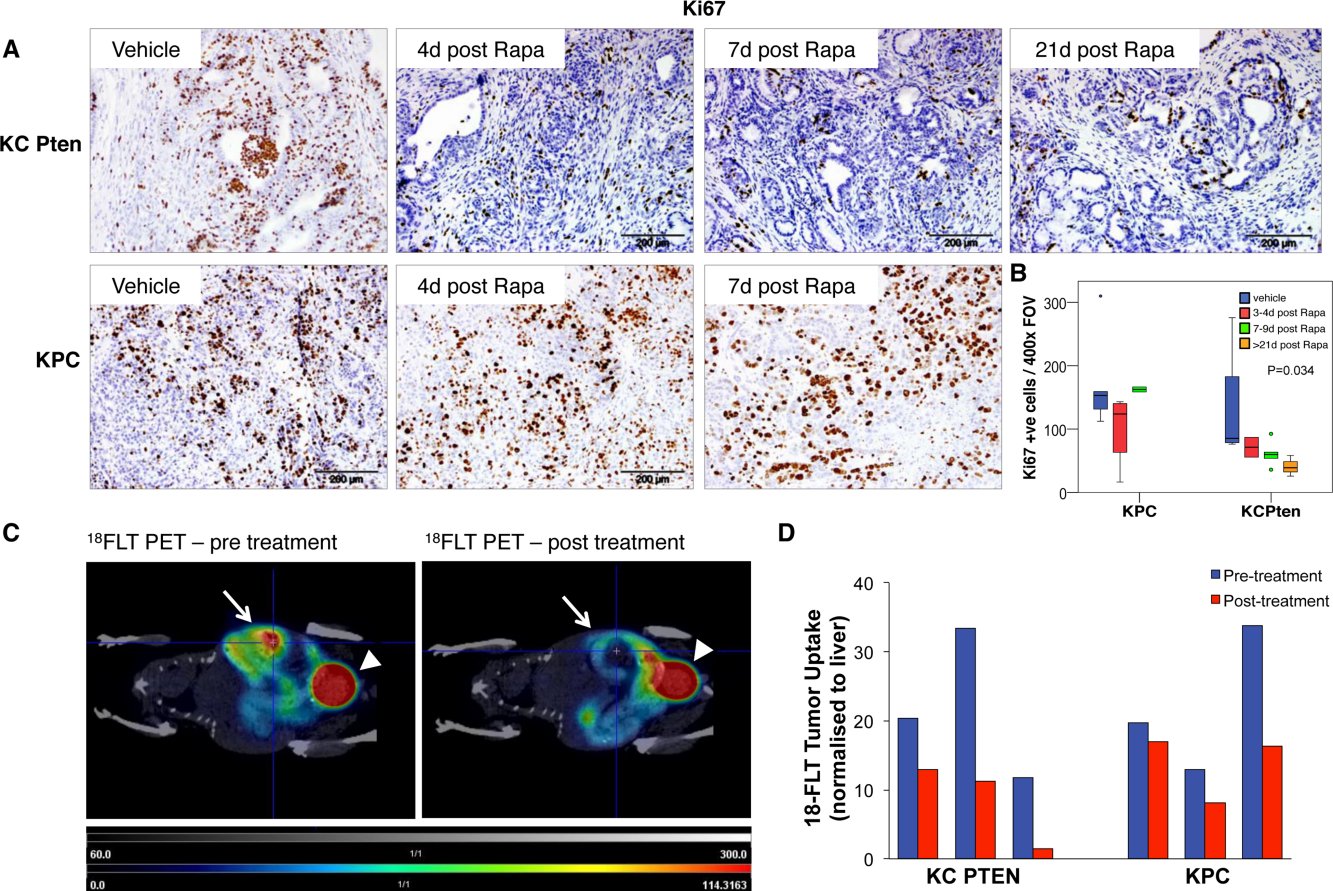
Mammalian target of rapamycin (mTOR) inhibition abrogates proliferation in Pten-deficient pancreatic ductal adenocarcinoma (PDAC). (A) Immunohistochemical staining for the proliferation marker Ki67 showing that rapamycin treatment results in a marked inhibition of proliferation in KC PTEN mice (upper panels), but not in KPC mice (lower panels). Sections from tumours harvested at the indicated time-points are shown here. (B) Graph showing quantification of the number of Ki67 positive cells per 400× field of view in sections from rapamycin or vehicle treated KC PTEN or KPC mice, as indicated. Ten fields were assessed per mouse, and at least 3 mice for each treatment group (blue=vehicle, red=3–4 days rapamycin, green=7–9 days rapamycin, orange=21+ days rapamycin). (C) Representative coronal plane ^18^F-3′-Fluoro-3′-deoxy-L-Thymidine (^18^FLT) positron emission tomography (PET)-CT images show the PET signal emitted from the pancreatic tumour (white arrows) as well as excreted tracer in the bladder (arrowheads) in a KC PTEN mouse at time of presentation (left panel), and after 4 days rapamycin treatment (right panel). (D) Graph of ^18^FLT uptake in KC PTEN and KPC tumours before, and following rapamycin treatment, based on maximum Standardised Uptake Value (SUV_Max_) in region of interest, and normalised to liver (n=3).

Given these findings, we hypothesised that the therapeutic efficacy of rapamycin in Pten-deficient tumours is achieved through growth arrest. We therefore assessed how rapamycin affected tumour cell proliferation by IHC for the proliferation marker Ki67. KC PTEN mice showed a marked reduction in the numbers of Ki67-positive cells following rapamycin treatment compared with control-treated mice (figure 3A, upper panels). There was a dramatic inhibition of proliferation 3–4 days post-treatment, and importantly, this inhibition continued with prolonged treatment and became significantly more pronounced in animals treated for more than 21 days (figure 3A upper panels, and figure 3B, p=0.034). There was a similar reduction in the number of Ki67-positive cells 3–4 days post-treatment in KPC mice, however, this effect was not sustained, and tumours from those animals that survived 7–9 days post-treatment showed the number of Ki67-positive cells was restored to a level similar to that seen in control animals (figure 3A lower panels, and figure 3B, p=0.465). When we examined p53 expression in rapamycin-treated KC PTEN mice we also found a significant increase in the number of p53-positive cells following treatment (see online supplementary figure S2A, p=0.050), consistent with this proliferative arrest. Since the lower proliferative index we observed in rapamycin-treated KC PTEN tumours coincided with histological change, we wanted to investigate whether changes in differentiation were responsible for the decrease in proliferation. We did not observe any changes in levels of amylase or cytokeratin 19, or the mucins MUC1, MUC2 or MUC5AC in rapamycin-treated KC PTEN tumours (see online supplementary figure 1). Nevertheless, we cannot completely rule out the possibility that the lower proliferative index is a consequence of the cystic phenotype, rather than the cause.

Since our data indicated that rapamycin treatment results in proliferative arrest, we wanted to measure this arrest in vivo, and also assess a potential biomarker of therapeutic efficacy. PET imaging has been used clinically and preclinically to evaluate therapeutic efficacy. In fact, PET imaging may be able to detect metabolic or proliferative changes earlier than the changes in tumour size that are detected by other imaging modalities.^26^ Thus, we performed PET imaging with ^18^FLT, a probe that marks cell proliferation,^27^ before and after rapamycin treatment in tumour-bearing KC PTEN and KPC mice. We observed a clear PET signal from the tumour in all mice imaged prior to treatment, confirming that these are highly proliferative tumours (figure 3C). Most exciting, however, was our finding that following treatment with rapamycin there was significantly reduced uptake of tracer in the tumours of all three KC PTEN mice (figure 3C,D). By contrast, we observed a marked reduction in uptake in only one out of the three KPC mice (figure 3D), and this one response may reflect the transient reduction in proliferation that we observed by IHC in KPC tumours, or the possibility that the tumour may have acquired a mutation affecting mTOR signalling. Consequently, we believe that ^18^FLT uptake (or lack of) may represent a promising functional biomarker that could be further developed for use as an indicator of antitumour efficacy in future clinical trials.

### mTOR inhibition with rapamycin acts primarily through S6 ribosomal protein

Although approved for some cancers, most clinical trials of rapalogues have been disappointing. Although this is likely due to a lack of patient selection, one reason frequently cited is that while rapamycin is very effective in targeting S6 kinase, it is less effective at targeting 4E-BP1.^28^ This was thought to be important as several studies suggested that 4E-BP1 is required for mTOR-mediated cell proliferation.^29–31^ Additionally, resistance to mTOR inhibition is reported to occur through loss of 4E-BPs or overexpression of eIF4E, suggesting that the 4E-BP1 response is critical.^31^ Further, incomplete inhibition of mTORC1-mediated phosphorylation of 4E-BPs, can lead to the activation of Akt via the loss of a negative feedback mechanism.^32–34^

**Figure 4:**
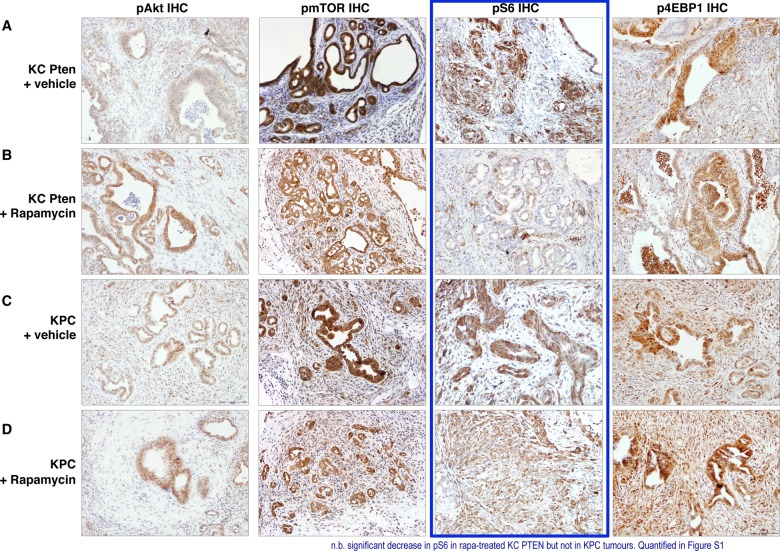
Mammalian target of rapamycin (mTOR) inhibition with rapamycin acts primarily through S6K. (A–D) Immunohistochemical analysis of pAKT, pmTOR, pS6 and 4EBP1 levels in vehicle and rapamycin treated KC PTEN and KPC tumours as indicated. Note the significant reduction in staining intensity of pS6 following rapamycin treatment in KC PTEN tumours, but not KPC tumours (highlighted in blue).

Thus, we examined the phosphorylation of downstream effectors of signalling through mTORC1: S6 ribosomal protein (as a read-out of S6K activity), and 4E-BP1. We also assessed expression of the phosphorylated form of mTOR itself, and of AKT (figure 4). As expected, tumours in KC PTEN exhibited very high levels of phosphorylated mTOR compared with KPC mice (figure 4, left inner panels and see online supplementary figure S2B). Interestingly, phosphorylation of AKT was slightly increased by treatment with rapamycin in tumours of either genotype (figure 4, left outermost panels and see online supplementary figure S2C), likely through loss of negative feedback on IRS1 from S6K1 and resulting increased mTORC2 activity, as has been described previously.^32–34^

Consistent with previously published work suggesting that S6K is the principal downstream signal affected by rapamycin, we observed decreased levels of pS6 in rapamycin-treated KC PTEN mice, but not in treated KPC mice (figure 4, right inner panels and see online supplementary figure S2D). By contrast, the levels of p4E-BP1 were not significantly altered by rapamycin in either genotype (figure 4, right outermost panels and see online supplementary figure S2E). Therefore, rapamycin appears to be exerting its antitumour effects though S6K, and thus, pS6 may be the best marker for measuring response to mTOR inhibitors. These data were supported by experiments in cell lines derived from KC PTEN and KPC tumours, in which rapamycin treatment resulted in a dramatic inhibition of phosphorylation of S6 (see online supplementary figure S3). Interestingly, rapamycin also blocked S6 phosphorylation in KPC cell lines, even though treatment did not significantly affect viability of KPC cells in vitro, suggesting that Pten-deficient cells are uniquely dependent on mTOR signalling.

### Low PTEN expression is associated with poor survival in human PDAC

**Figure 5:**
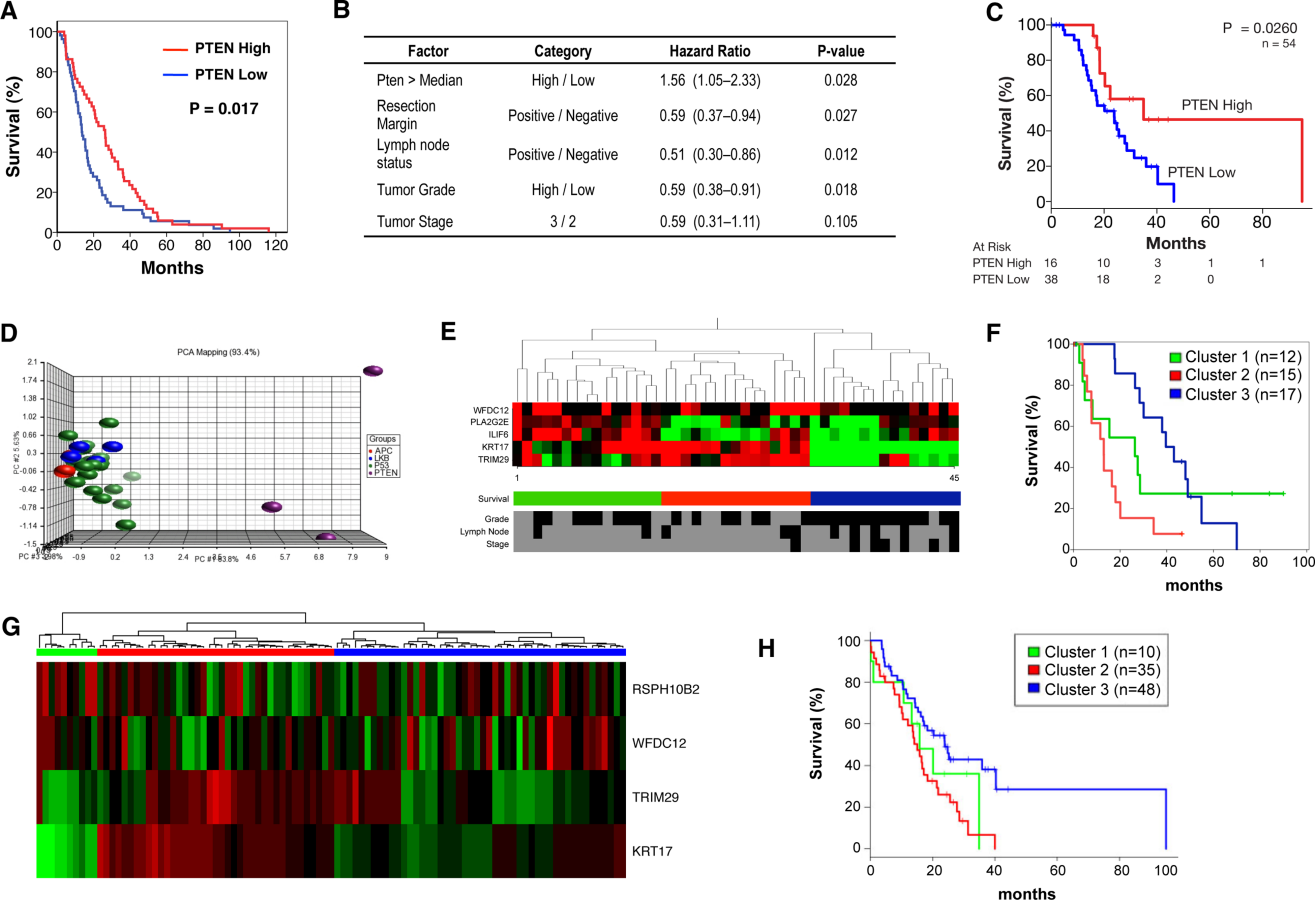
Low PTEN and expression of a low PTEN-associated signature predicts poor survival in human PDAC. (A) Kaplan–Meier analysis showing that cases with low Pten expression (n=59) have poorer outcomes compared with those with high expression (n=58, p=0.013), in the Glasgow cohort. (B) Table showing that by multivariate analysis, low PTEN expression is an independent predictor of survival. (C) Kaplan–Meier analysis showing that cases with low Pten expression (n=38) have poorer outcomes compared to those with high expression (n=16, p=0.026), in the Australian cohort as well. (D) Principal component analysis (PCA) of gene expression data generated from tumours in KC PTEN, KPC and *Pdx1-Cre*, *Kras^G12D/+^ Lkb1^fl/+^* and *Pdx1-Cre*, *Kras^G12D/+^ Apc^fl/+^* mice. This PCA was used to generate a gene expression signature specific to PTEN-deficient tumours. (E) Heat map showing that the PTEN-deficient signature could be used to delineate 3 groups of patients when applied to gene expression data from human PDAC patients (Glasgow cohort). Selected clinical data for the 45 patients is shown including tumour grade (low vs high) tumour stage (2 vs 3), lymph node involvement (negative vs positive). Black indicates low or negative, while grey indicates high or positive values. (F) Kaplan–Meier analysis showing human PDAC cases from the Glasgow cohort delineated on the basis of gene expression of low PTEN-associated signature. Cases with high expression of this signature (red, n=15) have significantly decreased survival compared to those with medium (green, n=15, p=0.1) or low expression (blue, n=15, p<0.0001, Log-Rank test). (G) Heat map showing validation of the PTEN-deficient signature used to delineate 3 groups of patients when applied to gene expression data from human pancreatic cancer patients (Australia cohort). (H) Kaplan–Meier curves showing difference of overall survival between 3 groups of patients identified by the PTEN-deficient signature in the Australia cohort (log-rank p=0.01).

We finally wished to determine which patients might show sensitivity to mTOR inhibition. Initially, we performed IHC for PTEN on a tissue microarray of resected human pancreatic tumour specimens. Expression was quantified using a histoscore method, and patients were divided into groups of low (n=59, mean histoscore 26.7) and high (n=58, mean histoscore 117.5) expression. Low PTEN expression was associated with significantly poorer survival in these patients (figure 5A, p=0.017). Furthermore, by multivariate analysis, low PTEN expression was an independent predictor of survival (figure 5B). These data were validated in a second group of patients in which low PTEN expression was again associated with significantly poorer survival (figure 5C, p=0.026).

We also wanted to assess whether the gene expression signature of KC PTEN tumours might define a subset of human PDAC. To identify a gene expression ‘signature’ specific to these mice, principal component analysis (PCA) was used to compare the transcriptome of these tumours with those arising in other mouse models of pancreatic cancer, notably KPC,^17^ ^18^ *Pdx1-Cre*, *Kras^G12D/+^ Lkb1^fl/+^*^20^ and *Pdx1-Cre*, *Kras^G12D/+^ Apc^fl/+^* mice (figure 5D). Importantly, the expression profile of the KC PTEN murine tumours was distinct from other tumours (figure 5D). We identified a signature of 219 probes that defined the KC PTEN phenotype and could be further refined to as few as eight probes. These mouse probes were then mapped to human microarray probes, and PCA analysis performed for each signature across two cohorts of human tumour samples.

In both cohorts, we were able to identify three distinct clusters of human pancreatic cancer using these signatures (figure 5E,G). Even when the smallest signature was used to cluster patients, this set of probes significantly correlated with poor survival in both cohorts (figure 5F,H). We also performed IHC to quantify protein expression of PTEN on a subset of tumours in the second cohort (n=46). These patients were used in an enrichment analysis whereby Fisher's exact test was used to test whether any of these gene expression clusters were enriched with a specific group of patients based on PTEN histoscore. Patients with low PTEN expression (histoscore ≤80, n=15) were more likely to be included in cluster 2 (red bars in figure 5G, p=0.009), importantly, the cluster with poorest survival. Thus, gene expression analysis of patient tumours, using a very small set of probes, may prove valuable as a method by which to identify patients with deregulated mTOR signalling, particularly where there is loss of function, but not of expression. Taken together, our data make a convincing case for the use of mTOR inhibitors in carefully selected human pancreatic cancer patients, and importantly, gene expression analysis might allow us to identify those patients.

## Discussion

Clinical trials of mTOR inhibitors in advanced pancreatic cancer have, thus far, have been preformed in unselected patients. We previously found that there is a subgroup of up to 20% of human PDAC in which increased activation of AKT/mTOR is associated with poor survival.^14^ And in sleeping beauty screens using the *Kras*-driven pancreatic cancer model, *Pten* ‘hits’ were the most common, reinforcing how important deregulation of mTOR might be in driving PDAC.^13^ ^14^ Here, using a preclinical mouse model of PTEN-deficient PDAC, we have shown that survival can be significantly extended using the classical inhibitor of mTORC1, rapamycin, and this is associated with a proliferative arrest. By contrast, there is little efficacy of rapamycin in the KPC model, typically used as a standard model of treatment-resistant PDAC. We believe that our data indicate that the KC PTEN model is exquisitely dependent upon signalling via mTOR, whereas the KPC model is not. These data demonstrate very well, preclinically at least, the value of the genotype-to-phenotype approach of targeting actionable phenotypes on the basis of genomic alterations.

Interestingly, a recent study found that activated PI3K signalling could phenocopy mutant *Kras* in a mouse model of pancreatic cancer, and concluded that KRAS acts through PI3K signalling to induce cancer.^35^ While the authors suggested that therapeutic targeting of PI3K signalling might be a promising approach for the treatment of pancreatic cancer, our data indicate that response to treatment will be dependent on the combination of genetic events in individual tumours. The antitumour effects of rapamycin are mediated primarily through S6K inhibition in our studies. The recent report that KRAS-induced pancreatic cancer was substantially reduced in mice expressing an S6 mutant that could not be phosphorylated also highlighted the importance of this signalling arm downstream of mTOR.^36^ Inhibition of mTOR, and thus S6 in our model led to proliferative arrest. Others have found that the 4E-BP proteins mediate mTOR-driven proliferation,^29^ ^37^ however, we did not observe any effects on 4E-BP1 phosphorylation following rapamycin treatment. New research has shown that mTOR signals through S6 to stimulate de novo pyrimidine synthesis, and thus, control cell proliferation,^38^ ^39^ and inhibition of this process may be the mechanism by which proliferation is blocked in our mice.

This proliferative arrest may provide a useful biomarker of therapeutic activity in clinical trials. Using a clinically relevant imaging modality and tracer, we were able to visualise this proliferative block days after commencing treatment, and it is not unreasonable to think that this approach might be used in the clinic. Pilot studies have already found that ^18^FLT and ^18^FDG PET imaging can be used to predict therapeutic responses in cancer patients,^40^ ^41^ and this might be particularly important in pancreatic cancer where repeat access to tissue can be limiting.

To date, human trials involving rapalogues have been performed in patients with advanced disease, and with no selection to identify tumours that might be particularly dependent upon mTOR signalling. Our data suggest that, at least in resected cases, only 20% of patients have aberrant activation of this pathway. More germane, then, are the studies of exceptional responders: most notably, a response to mTOR inhibition in a Peutz–Jeghers patient,^6^ and also the trial of an AKT inhibitor in which a patient with metastatic pancreatic cancer with known PTEN loss exhibited a marked response.^7^ Both these reports nicely illustrate the phenotype-to-genotype approach to targeted therapy.

Finally, our findings illustrate that targeted therapies are effective with appropriate selection, and highlight the need for more trials that test personalised therapies in patients where there is a clear actionable phenotype. Preclinical models will be extremely valuable for testing first-line targeted therapies, and also for determining mechanisms of resistance, and assessing follow-up or combination treatments.

## Supplementary Material

Web supplement

Web figures
