# Supplementary material for: Targeting mTOR dependency in pancreatic cancer
Source: Gut. 2014 Apr 9;63(9):1481–9. doi: 10.1136/gutjnl-2013-306202 (PMC4145424; doi:10.1136/gutjnl-2013-306202)
Supplement: Web supplement [file gutjnl-2013-306202-s1.pdf]

SUPPLEMENTARY METHODS

^{18}F-FLT Synthesis

^{18}F-FLT was produced via a modification of Lee *et al*[1], using 3-N-Boc-5'-O-dimethoxytrityl-3'-O-nosylthymidine as a precursor. Briefly, $^{18}F^-$ was produced from a cyclotron (PET RPU, Gartnavel General Hospital, Glasgow). A solution of 10µl tetrabutylammoniumbicarbonate ($TBAHCO_3$) was added to $^{18}F^-$ (500MBq) in approx. 30µl of H_2O. The solution was dried under a stream of argon before adding 5 mg of precursor in a solution of 100µl of CH_3CN and 200µl of t-BuOH. The reaction time and temperature for ^{18}F-fluorination was 5min at 110°C. After ^{18}F-fluorination, the solvent was removed under a stream of argon and added was 500µl of 1N HCl and 100µl of CH_3CN for de-protection at 110°C for 5 min. The solution was neutralized by addition of 250µl of 2N NaOH. Purification and analysis was achieved using reversed-phase high-performance liquid chromatography (HPLC). The solvent was removed by rotary evaporation and the ^{18}F-FLT was reconstituted in saline for intravenous administration.^{18}F-FLT was produced with an end of synthesis yield of between 15-31% (n=6) and the radiochemical purity was greater than 99%.

^{18}F-FLT PET-CT Imaging and Quantitation

Static PET acquisitions were performed for 1hr and reconstructed using the MLEM algorithm with 12 iterations. CT data was acquired for 12 minutes in high-resolution mode (600 projections) using the 45kV and 0.2mA settings. Fused PET-CT images were created and PMOD software (PMOD Technologies Ltd, Zurich, Switzerland) used to perform qualitative and quantitative assessments of the data.

A qualitative assessment of the changes in FLT uptake before and after treatment was performed by scaling relevant coronal sections to the same value of the percentage of injected

dose/ml. Changes in uptake were quantified by calculating maximum Standardized Uptake Value (SUV_{Max}) within the tumour and dividing by SUV_{Mean} of normal reference tissue (liver), using the same volume of interest applied to pre- and post-treatment images.

Human Gene Expression Enrichment Analysis (Glasgow)

Tumour samples were prospectively acquired and processed from 48 patients with histologically confirmed non-pre-treated pancreatic ductal adenocarcinoma resected with curative intent by pancreaticoduodenectomy from the West of Scotland Pancreatic Unit, Glasgow Royal Infirmary as previously described[2]. RNA was extracted and processed as previously described[2] and subsequently arrayed on Agilent 44K whole genome microarrays to assess gene expression as previously described[3]. An average value of the replicate spots for each mRNA was normalized and uploaded into Biometric Research Branch (BRB)-ArrayTools 3.9[4]. Clustering analysis was performed according to the PTEN-deficient signature. Kaplan–Meier survival analysis was used to analyse overall survival time from date of resection. Patients alive at the follow-up point were censored. Survival analysis was performed, using a log-rank test, according to PTEN-deficient signature subgroup to determine their influence on outcome.

Human Gene Expression Enrichment Analysis (Australia)

The gene expression data is from The International Cancer Genome Consortium (ICGC) pancreatic cancer project. Samples used were prospectively acquired and processed as previously described[5], and restricted to primary operable, non-pre-treated pancreatic ductal adenocarcinoma (for full protocol see APGI website[6]). The project included gene profiling of 97 confirmed PDAC primary tumour samples using Illumina HumanHT-12 v4 microarray, 93 of which were used in the survival analysis. The same method described in the previous

paper[5] was applied for the expression data analysis and survival analysis. Gene expression clusters were identified using the human orthologs of the mouse 8-gene signature, while 6 genes have probes on HumanHT-12 v4 microarray and 4 genes have significant expression different from background noise. Out of 93 patient tumours, 46 tumours were also scored for Pten expression by IHC (as described above). 3 cores per tumour were scored, and the median value used to represent Pten activity in each patient tumour. These 46 patient tumours were dichotomized into two groups based on Pten expression level. Two-sided Fisher's exact test was applied for each of 3 gene expression clusters to determine whether patients of a gene expression cluster were statistically enriched in Pten IHC dichotomized groups.

REFERENCES

1 Lee SJ, Oh SJ, Chi DY, et al. Simple and highly efficient synthesis of 3'-deoxy-3'-[18f]fluorothymidine using nucleophilic fluorination catalysed by protic solvent. Eur J Nucl Med Mol Imaging 2007;**34**:1406-9.

2 Jamieson NB, Morran DC, Morton JP, et al. Microrna molecular profiles associated with diagnosis, clinicopathologic criteria, and overall survival in patients with resectable pancreatic ductal adenocarcinoma. Clinical cancer research : an official journal of the American Association for Cancer Research 2012;**18**:534-45.

3 Dozynkiewicz MA, Jamieson NB, Macpherson I, et al. Rab25 and clic3 collaborate to promote integrin recycling from late endosomes/lysosomes and drive cancer progression. Dev Cell 2012;**22**:131-45.

4 http://linus.Nci.Nih.Gov/brb-arraytools.Html

5 Biankin AV, Waddell N, Kassahn KS, et al. Pancreatic cancer genomes reveal aberrations in axon guidance pathway genes. Nature 2012;**491**:399-405.

6 http://www.Pancreaticcancer.Net.Au/
