# Supplementary material for: Targeting mTOR dependency in pancreatic cancer
Source: Gut. 2014 Apr 9;63(9):1481–9. doi: 10.1136/gutjnl-2013-306202 (PMC4145424; doi:10.1136/gutjnl-2013-306202)

# Supplementary Figures

## Supplementary Figure 1

**Cyst formation in rapamycin-treated KC PTEN tumors is not accompanied by changes in markers of differentiation.** A-B) Immunohistochemical analysis of amylase, cytokeratin 19, MUC1, MUC2 and MUC5AC expression in (A) vehicle and (B) rapamycin-treated KC PTEN tumours. No major changes were observed for any of these markers.

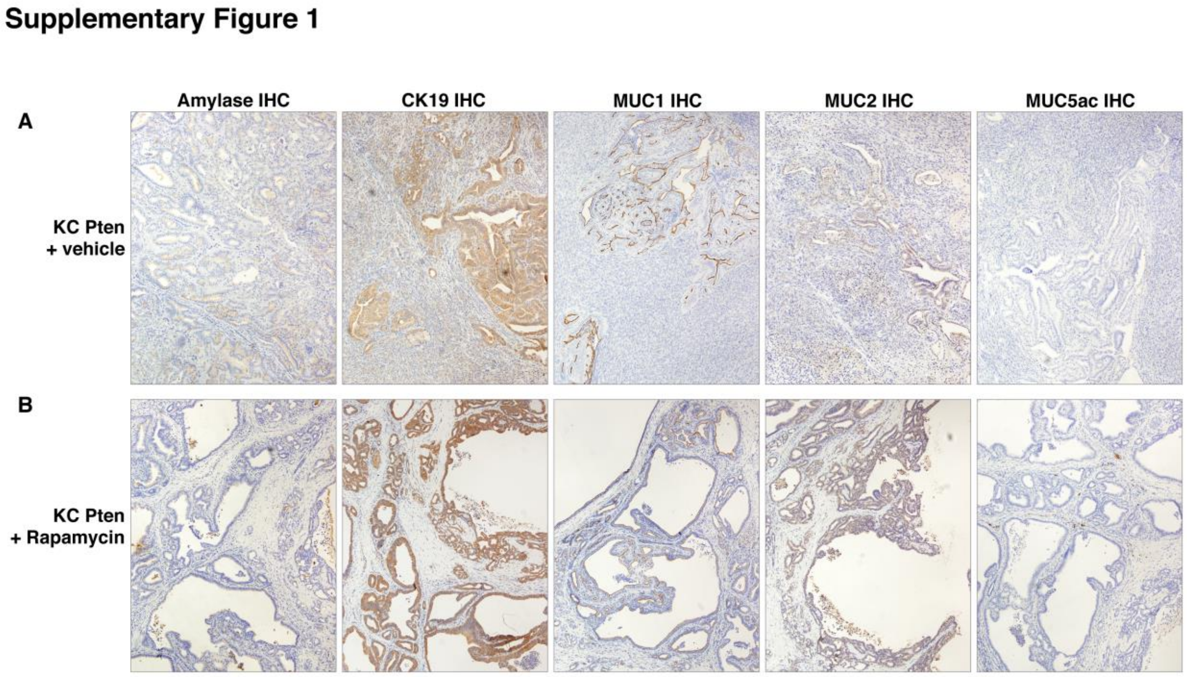


## Supplementary Figure 2

**Rapamycin treatment induces p53 and inhibits S6 ribosomal protein activity in PTEN-deficient tumors.** A) Boxplot showing quantification of the number of p53-positive cells per 400x field of view in sections from rapamycin treated (red bars) or vehicle treated (blue bars) KC PTEN mice, as indicated. B) Boxplot showing quantification of the intensity (as histoscore) of pmTOR staining in sections from rapamycin treated (red bars) or vehicle treated (blue bars) KC PTEN or KPC mice, as indicated. C) Boxplot showing quantification of the intensity (as histoscore) of pAKT staining in sections from rapamycin treated (red bars) or vehicle treated (blue bars) KC PTEN or KPC mice, as indicated. D) Boxplot showing quantification of the intensity (as histoscore) of pS6 staining in sections from rapamycin treated (red bars) or vehicle treated (blue bars) KC PTEN or KPC mice, as indicated. E) Boxplot showing quantification of the intensity (as histoscore) of

p4EBP1 staining in sections from rapamycin treated (red bars) or vehicle treated (blue bars) KC PTEN or KPC mice, as indicated. At least 4 full face sections were scored for each group.

**Supplementary Figure 2**

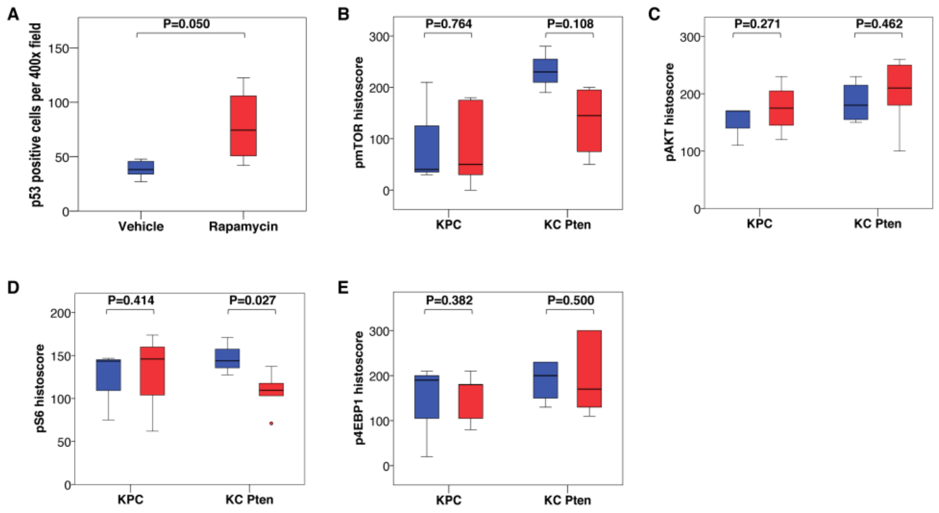


## Supplementary Figure 3

**Rapamycin treatment strongly inhibits activation of S6 ribosomal protein in PDAC cell lines.** PDAC cell lines established from tumors arising in KC PTEN and KPC mice (n = 3 per genotype), were treated with rapamycin at a concentration of 10µM for 2 hours, and then lysates prepared. Immunoblotting was performed for phospho- and total AKT, phospho- and total mTOR, phospho- and total S6 ribosomal protein, and β-actin as a loading control.

**Supplementary Figure 3**

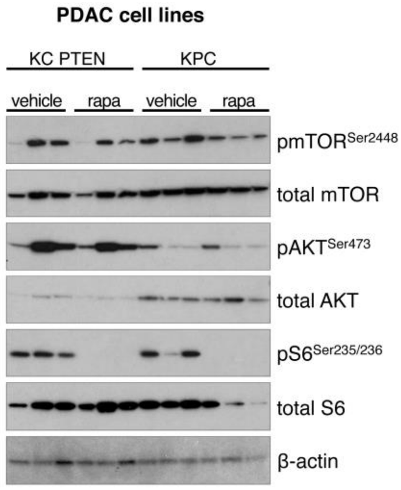

Supplement: Web figures [file gutjnl-2013-306202-s2.pdf]
